# Dynamic Evolution of Fibroblasts Revealed by Single-Cell RNA Sequencing of Human Pancreatic Cancer

**DOI:** 10.1158/2767-9764.CRC-23-0489

**Published:** 2024-12-02

**Authors:** Slavica Dimitrieva, Jon M. Harrison, Jonathan Chang, Michelle Piquet, Mari Mino-Kenudson, Millicent Gabriel, Vivek Sagar, Heiko Horn, Kasper Lage, Julie Kim, Gang Li, Shaobu Weng, Cynthia Harris, Anupriya S. Kulkarni, David T. Ting, Motaz Qadan, Peter J. Fagenholz, Cristina R. Ferrone, Angelo L. Grauel, Tyler Laszewski, Alina Raza, Markus Riester, Tim Somerville, Joel P. Wagner, Glenn Dranoff, Jeffrey A. Engelman, Audrey Kauffmann, Rebecca Leary, Andrew L. Warshaw, Keith D. Lillemoe, Carlos Fernández-del Castillo, David A. Ruddy, Andrew S. Liss, Viviana Cremasco

**Affiliations:** 1 Oncology Data Science, Novartis Biomedical Research, Basel, Switzerland.; 2 Department of Surgery, Massachusetts General Hospital, Harvard Medical School, Boston, Massachusetts.; 3 Oncology Translational Research, Novartis Biomedical Research, Cambridge, Massachusetts.; 4 Oncology Innovative Targets and Technologies, Novartis Biomedical Research, Cambridge, Massachusetts.; 5 Department of Pathology, Massachusetts General Hospital, Harvard Medical School, Boston, Massachusetts.; 6 Oncology Data Science, Novartis Biomedical Research, Cambridge, Massachusetts.; 7 Mass General Cancer Center, Boston, Massachusetts.; 8 Oncology, Novartis Biomedical Research, Cambridge, Massachusetts.

## Abstract

Cancer progression and response to therapy are inextricably reliant on the coevolution of a supportive tissue microenvironment. This is particularly evident in pancreatic ductal adenocarcinoma, a tumor type characterized by expansive and heterogeneous stroma. Herein, we employed single-cell RNA sequencing and spatial transcriptomics of normal, inflamed, and malignant pancreatic tissues to contextualize stromal dynamics associated with disease and treatment status, identifying temporal and spatial trajectories of fibroblast differentiation. Using analytical tools to infer cellular communication, together with a newly developed assay to annotate genomic alterations in cancer cells, we additionally explored the complex intercellular networks underlying tissue circuitry, highlighting a fibroblast-centric interactome that grows in strength and complexity in the context of malignant transformation. Our study yields new insights on the stromal remodeling events favoring the development of a tumor-supportive microenvironment and provides a powerful resource for the exploration of novel points of therapeutic intervention in pancreatic ductal adenocarcinoma.

**Significance:**

Pancreatic cancer remains a high unmet medical need. Understanding the interactions between stroma and cancer cells in this disease may unveil new opportunities for therapeutic intervention.

## Introduction

The tumor microenvironment is a complex aggregate of cell communities that collectively support the nascent tumor, fostering aggressive cancer cell behaviors, immunologic failure and, ultimately, tumor progression (1). Fibroblasts, in particular, play a crucial role in this ecosystem by producing various factors that directly support malignant cells (2, 3) and indirectly reprogram tumor immunity (4–6) and tissue structural features (7–13). In this context, therapeutic agents directed toward fibroblasts may identify stromal abnormalities in the tumor microenvironment, ultimately restraining cancer progression (4, 5, 14–19). However, such approaches remain limited because of our poor understanding of the diversity and dynamics of tumor stromal cells in their natural context.

The recognized role of fibroblasts in cancer progression is broadly applicable to solid tumors, but it is of even more critical importance in indications such as pancreatic ductal adenocarcinoma (PDAC), an aggressive malignancy widely recognized for its expansive and heterogeneous stromal component (19, 20). Understanding the interplay between stroma and cancer cell populations may therefore provide insight into programs underlying tumor evolution in this disease. From this perspective, the application of single-cell technologies can greatly aid in shedding light into the complexity of the cellular elements and interactions in PDAC, paving the way for the discovery of new points of intervention for disease management. Such approaches have already provided an enhanced resolution of the stromal landscape in tumors, generating valuable insights onto the wide-ranging array of phenotypes and functions of fibroblasts in the tumor microenvironment (7, 21–26). Still, availability of fresh patient samples from relevant disease settings and associated controls, as well as tissue processing methodologies for examining stromal cells without compromising unique or rare cell populations, continues to be obstacles that hinder interpretation of some of these emerging insights.

In this study, we aimed to generate a robust and representative dataset that captures stromal dynamics within the human pancreas. Normal pancreatic tissue as well as tissue samples from patients with pancreatitis, treatment-naïve PDAC, and PDAC treated with standard-of-care FOLFIRINOX-based neoadjuvant therapy (NAT) were analyzed using single-cell and spatial transcriptomic (ST) technologies to infer the complex interdependence of tumor cells and their stromal microenvironment. These data allowed us to uncover the temporal and spatial coevolution of tumor cells and stroma, highlighting the cellular circuitries governing tissue remodeling in PDAC. Ultimately, this dataset represents an invaluable resource to illuminate the cell populations and microenvironmental cues that support PDAC progression and identify novel molecular targets for more effective cancer treatments.

## Materials and Methods

### Patient sample identification, collection, and processing

Following Institutional Review Board approval, patients undergoing pancreatectomy for treatment-naïve PDAC, FOLFIRINOX-based NAT therapy–treated PDAC, and pancreatitis at the Massachusetts General Hospital between June 2019 and September 2020 were identified for tissue sample collection (Supplementary Table S1). Normal pancreas was collected from patients undergoing pancreatectomy for mucinous cystic neoplasm, taken from a portion of the surgically resected pancreas that was distant from the mucinous cystic neoplasm and was confirmed by histologic analysis to have normal pancreatic architecture (no signs of inflammatory injury or malignancy). Pancreatitis samples were collected from patients with radiographic evidence of chronic pancreatitis and underwent pancreatectomy for recurrent pain episodes. These included “typical” toxic (alcohol) and extrapancreatic (biliary and post-operative) etiologies as well as a sample of type I autoimmune pancreatitis. Immediately post-resection, a minimum of 50 mg of tissue was collected from the tumor core after histologic verification for the presence of cancer cells. For benign samples, tissue was collected from an area of pancreas suitable for procurement. Tissue samples were then enzymatically disassociated in RPMI containing 0.1 mg/mL DNase I (Roche), 0.2 mg/mL Collagenase P (Roche), 0.1 mg/mL Dispase (Gibco, Thermo Fisher Scientific), and 2% FBS following a protocol we have previously described and validated for tissue processing and recovery of stromal components without prior cell enrichment (27–29). Written informed consent was obtained from all patients included in this study; studies were conducted in accordance with ethical guidelines and approved by MGH review board.

### Single-cell RNA sequencing and data preprocessing

The 10x Genomics Chromium Single Cell 3′ Reagents v3 kit (10x Genomics) was used with standard conditions and volumes to process cell suspensions for 3′ transcriptional profiling. The cell suspension volumes were calculated for a target cell recovery of between 4,000 and 8,000 cells for all disassociated samples and loaded on the Chromium according to manufacturer’s guidelines. The resultant-purified cDNAs were quantified on an Agilent TapeStation (Agilent) using High Sensitivity D5000 ScreenTapes and Reagents. The final single cell 3′ libraries were quantified using an Agilent TapeStation using High Sensitivity D1000 ScreenTapes and Reagents. The libraries were then diluted to 10 nmol/L in Qiagen elution buffer, denatured, and loaded on an Illumina MiSeq at 6 μmol/L with the MiSeq Reagent Kit v3 (Illumina) to access sample quality and loading normalization for the HiSeq4000. The normalized libraries were loaded at 160 pmol/L on an Illumina cBOT and sequenced on a HiSeq4000 for 28 base pairs on the first read, followed by an eight bp index read, and a 91 bp second read, using two HiSeq4000 SBS kits, 50 cycles. All sequence intensity files were generated using the Illumina Real Time Analysis software. The resulting intensity files were demultiplexed and then aligned to the human genome, version hg38, using the 10x Genomics CellRanger v3.0.1 software package.

### Single-cell RNA sequencing data quality control and processing

All computational analyses and visualizations were performed in R v3.6.3. The Seurat package (v3.2.2) was used for quality control and downstream analysis of single cell RNA sequencing (scRNA-seq) data (30). The ambient mRNA profile of each library was determined based on the content of empty droplets for that library. The SoupX algorithm was then used to estimate and remove ambient mRNA contamination from the original expression matrix, as described in the tutorial (https://github.com/constantAmateur/SoupX; ref. 31). For each scRNA-seq library, CellRanger filtered count matrices were processed using the standard Seurat pipeline and were clustered at a relatively low resolution (0.2). This clustering was then passed to SoupX. The ambient mRNA contamination fraction was estimated using the *estimateNonExpressingCells* and *calculateContaminationFraction* function. Exocrine (*PRRS1*, *PNLIP*, *CLPS*, and *CTRB*) or endocrine and immune (*INS* and *IGKC*) cell marker genes used for estimating the contamination fraction for libraries coming from nonmalignant and malignant samples, respectively. The *adjustCounts* function was used to correct the expression matrices. The corrected count matrices were then analyzed using the Seurat workflow. Low quality cells with either <200 expressed genes, >6,000 expressed genes, or >30% mitochondrial content were excluded from the dataset. Count matrices were merged and log-normalized using a scaling factor of 10,000. Principal component (PC) analysis was performed using 2,000 highly variable genes. To select the appropriate number of PCs, the Elbow plot heuristic was used. A shared nearest neighbor graph was built with the first 20 PCs using Seurat’s *FindNeighbors* function, and the cells were clustered with a Louvain algorithm with *FindClusters*. A high resolution of 1 was selected to generate a large collection of cell clusters to capture distinct cell lineages even if present at low proportions. Cluster markers were identified using Seurat’s *FindAllMarkers* function. After manual inspection of all clusters, doublet clusters were identified based on observation of markers from more than one cell type and were removed. Finally, the Uniform Manifold Approximation and Projection for Dimension Reduction method was used to plot the dimensionally reduced dataset. The *R*-based *Clustree* package, which compares cell groups from related PCs, helped determine the appropriate resolution for PC interpretation (32).

### Differential gene expression and pathway enrichment analyses

Differential gene expression analyses were performed on cells with a minimum of 10% feature expression and average log-fold change above 0.25 as part of the Seurat workflow. Pathway enrichment analyses were performed with the ClusterProfiler package (v3.14) using Kyoto Encyclopedia of Genes and Genomes (KEGG) and Reactome pathway annotations (33–35). Pathway enrichment was computed for all cluster marker genes with avg_logFC > 0.5 and adjusted *P* value < 0.01 using the *enrichKEGG* and *enrichPathway* functions. Selected significantly enriched pathways (with adjusted *P* value < 0.05) were visualized using *ggplot2*.

### Clustering and analysis of ductal and cancer cells

Ductal and cancer cells were subsetted from the full dataset and re-clustered using five PCs at resolution 0.1. The optimal number of PCs was determined based on the Elbow heuristic. Marker genes for each cluster were inferred using the *FindAllMarkers* function of Seurat. The cancer lineage was additionally subclustered alone using five PCs at resolution 0.1. For each cancer cell, transcriptional subtype scores were determined using the *AddModuleScore* function based on previously defined genes (36).

### Clustering and trajectory inference of fibroblast cells

Fibroblast cells were subsetted from the full dataset and re-clustered using five PC at resolution 0.2. Cell trajectories were inferred using Slingshot (v1.4; ref. 37), setting cluster “F1” as starting point. Gene expression changes over the estimated trajectory leading to cancer-associated fibroblasts (CAF) were visualized by fitting a generalized additive model with smoothing spline regression, using the R package mgcv (v1.8.31). RNA velocity analysis for the fibroblast lineage was performed using the Python package scVelo (38). For each library, the matrix for spliced and unspliced transcripts was constructed using kallisto-bustools (39). Subsequently, RNA velocity was calculated using the generalized dynamical model, following the official scVelo tutorial. Finally, the cell trajectories in the fibroblast lineages were visualized with the Uniform Manifold Approximation and Projection for Dimension Reduction embeddings from Seurat.

### Cell–cell interaction analysis

Cell–cell interaction analyses from scRNA-seq data were performed using two different R-based intercellular communication tools: CellChat (v1.1.3) and NicheNet (v1.0.0; refs. 40, 41). For each sample type, CellChat analyses were run with individual fibroblast subpopulations and with all fibroblast subpopulations combined. Normalized count matrices along with cell annotation metadata were processed through the standard CellChat pipeline using the CellChatDB human ligand–receptor interaction database. Default values for all parameters were used, except that the communication probability was calculated with a truncated mean of 10%. The minimum number of cells required in each cell group for cell–cell communication inference was set to 100. The interaction graphs were produced using the *netVisual_circle* function. The analysis of dominant sender and receiver cell populations and their visualization in a two-dimensional space was done using *netAnalysis_signalingRole_scatter*. The *netVisual_aggregate* function was used to infer and visualize the communication network of the individual signaling pathways. Additionally, the *nichenetr* package was used to examine the intercellular communication between ligands and gene sets of interest in untreated patients with PDAC. Activity of the ligand was established by correlation with changes in the expression of target genes, as compared with background genes, using Pearson correlation coefficient. Specifically, the ligands released from any cell population with respect to the receptors present on cancer cells, CAFs, and transitional fibroblasts were assessed. The ligand–target matrix (ligand_target_matrix.rds), ligand–receptor network (lr_network.rds), and weighted networks (weighted_networks.rds) were downloaded from Zenodo (https://zenodo.org/record/3260758). For the ligand–receptor network, the following databases were used: “kegg,” “guide2pharmacology,” and “ramilowski.” Genes were considered expressed in a cell population if they had nonzero counts in at least 30% of cells in that cell population and were filtered to keep only those present in the ligand–receptor network. Two gene sets of interests were defined: (i) differentially expressed (DE) genes comparing cancer to normal ductal cells in untreated patients with PDAC and (ii) DE genes comparing CAFs and transitional fibroblasts to normal fibroblasts in untreated patients with PDAC. In this analysis, the threshold for differential expression was set to avg_logFC > 0.75 and adjusted *P* value < 0.01. The ligand activity for the genes present in the ligand–target matrix was calculated using the *predict_ligand_activities* function. Pearson correlation was used to rank genes, and circos plots were used to visualize the top-ranked ones using the *chordDiagram* function.

### Master transcriptional regulator analysis

To infer the transcription factor regulatory network in fibroblasts, we used all 1,639 human transcription factors (42). We first performed regulatory network analysis for the five types of fibroblasts separately, each with corresponding expression data including one type of fibroblast and the rest of fibroblasts, using the ARACNe-AP software (43). Next, we performed master regulator analysis using the ssmarina package (https://figshare.com/articles/dataset/ssmarina_R_system_package/785718), a modification of the MAster Regulator INference algorithm (44). Enrichment of the predicted targets was assessed by comparing the gene expression between two groups. Regulators with FDR-corrected *P* values below 0.01 were inferred as candidate master regulators between two given groups.

### ST data generation

Spatial gene expression data were captured using 10x Genomics for formalin-fixed, paraffin-embedded (FFPE) samples. FFPE tissue blocks were sectioned, and tissue was placed directly onto a 10x Genomics Visium Spatial slide. The slide was incubated for 3 hours at 42°C and dried overnight in a desiccator. The tissue was then prepared according to the manufacturer’s instructions where the tissue was deparaffinized, hematoxylin and eosin–stained, imaged, and decrosslinked. The whole-transcriptome probe panel was then added to the tissue. The ligated products are released from the tissue and captured on the Visium slides where extension takes place and generates spatially barcoded product that can then proceed with library preparation. Final libraries were sequenced on an Illumina NovaSeq instrument using recommended read lengths and sequencing depths.

### ST data processing

Visium ST reads were mapped against the human genome (GRCh38), and Ensembl (release 85) gene transcripts were quantified using the *spaceRanger* pipeline (v 1.3.0). The Seurat package (v3.1.4) was used to process spot by gene expression data. For each ST sample, spots with <500 genes detected were filtered out. Expression data were normalized using the SCTransform method, regressing out the number of genes detected in each spot (nFeature_Spatial; ref. 45). Dimensionality reduction and clustering of the ST expression data were done using the first 30 PCs. A shared nearest neighbor graph was built using Seurat’s *FindNeighbors* function, and the spots were clustered applying the Louvain algorithm with resolution of 1. ST data were then used to establish tissue locations of the cell types presented. To map the scRNA-seq labels to individual ST slides, the anchor-based integration workflow introduced in Seurat v3 was followed, using the scRNA-seq data as ref. 30. For this purpose, the scRNA-seq data were reprocessed and renormalized using the *SCTransform* method, learning a noise model on 3,000 cells. Next, for each ST sample, the *FindTransferAnchors* and *TransferData* functions were applied to perform cell type label transfer, setting the normalization method to SCT. PC analysis on the ST data using the first 30 PCs was used as dimensionality reduction for the weighting anchors. Prediction scores for each spot for each cell type were computed and visualized using the *SpatialFeaturePlot* function. Of note, similar cell annotation outputs were obtained using alternative algorithms (e.g., Giotto, CellTrek), supporting validity of the spatial outputs generated with Seurat.

### RNA-ISH

RNA-ISH was performed using RNAscope 2.5 LS Duplex Reagent Kit [Advanced Cell Diagnostics (ACD)] on the BOND RX 6.0 platform (Leica Biosystems Inc.) according to the manufacturer’s instructions. Briefly, 5 μm sections of FFPE tissue mounted on Superfrost Plus Microscope Slides (Thermo Fisher Scientific) were baked for 1 hour at 60°C and placed on the BOND RX for processing using the ACD Red/Brown Duplex Protocol. Probe cocktails containing ACD RNAscope probes specific for *SCGB3A1*, *MUC5B*, and *CRISP3* or *FXYD3* and *SLCA4* were used in a final 2-hour probe hybridization step. Samples were counterstained with hematoxylin. The target mRNAs were then visualized using a standard brightfield microscope which showed the *FXYD3 and SLCA4* probe cocktail signal as red and the *SCGB3A1*, *MUC5B*, and *CRISP3* probe cocktail signal as brown.

### KRAS assay

A nested PCR reaction scheme was employed to first amplify the mutational region of KRAS encompassing codons 12 through 61 (nest 1), and then to add Illumina flow cell binding sequences (nest 2). cDNA generated from the amplification step of the 10x Genomics Single Cell 3′ library construction was used as the starting material for nest 1 PCR amplification. Input cDNA material of 10 ng of was used, and all samples were run in duplicate in 25 μL reaction volumes. Thermal cycling parameters and primer design can be found in Supplementary Table S2. Following nest 1 amplification, AMPure XP bead purification was used to remove unused primer and nonamplified cDNA. The purified elutes were then used as input for nest 2 amplifications. The nest 2 amplicons were purified of excess primer using AMPure XP bead purification and quantified using Agilent’s DNA TapeStation system. Purified amplicons were then loaded onto an Illumina Miseq per manufacturer’s specifications. To detect KRAS-mutant variants for a given 10x cellular barcode, a k-mer–based search method was applied using 38-mer sequences as “fishhooks” to capture specific variants. A seed match (6-mer) and extension algorithm were used to identify the occurrence of each fishhook in a 10x cell, allowing for a maximum of two base mismatches after a perfect seed match. Simultaneously, unique molecular identifier **(**UMI) sequences (transcripts) associated with these variants were also quantified and filtered if they had less than five reads coverage or contained both wildtype and mutant sequences. The UMI occurrence information was then used to assign wildtype or mutant status to a 10x cell, considering a variant assignment to be confident if it was based on at least two UMIs.

### Data availability

The sequencing data that support the findings of this study have been deposited (dbGaP Study Accession: phs003751.v1.p1). All remaining data related to the study are included in the article and Supplementary Materials.

## Results

### A bird’s-eye view of the pancreas microenvironment

**Figure 1:**
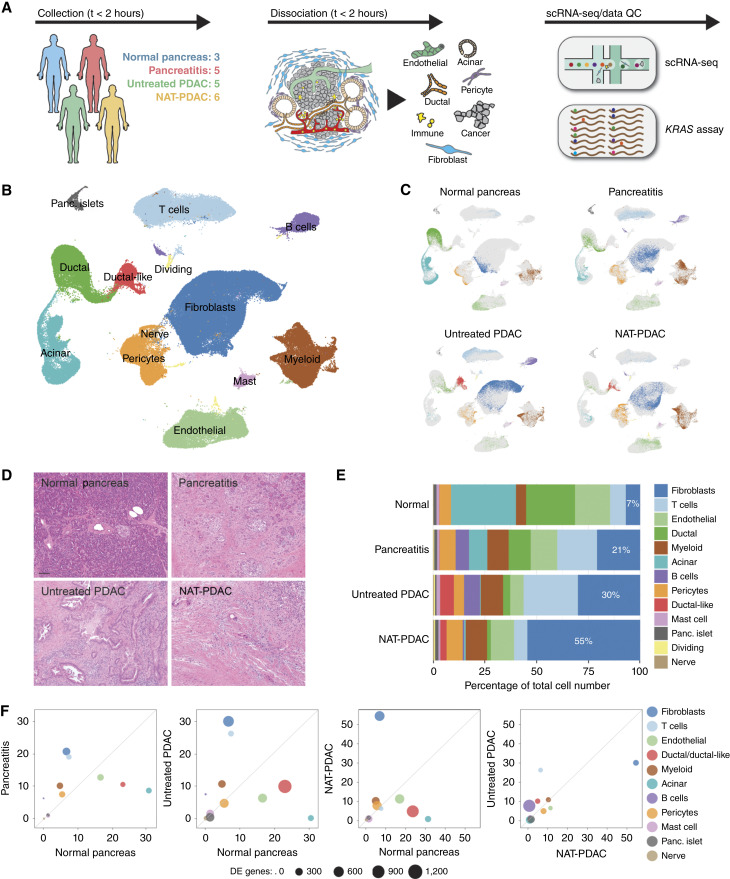
scRNA-seq of human pancreatic tissues. **A,** Schematic representation of the types of tissues employed in this study and the experimental pipeline for their analysis by scRNA-seq. **B,** UMAP visualization of single cells from all samples colored by cell type. **C,** UMAP projections of single cells from each tissue type (colored) contributing to the global UMAP (gray). **D,** Representative images of H&E-stained sections of each tissue type. Scale bar, 100 μm. **E,** Quantification of cell types within each tissue type. **F,** Quantification of cell populations (% from the total) along with the number of DE genes in each cell population between tissue types. UMAP, Uniform Manifold Approximation and Projection for Dimension Reduction; H&E, hematoxylin and eosin.

To explore the complex cellular networks in the PDAC microenvironment, we sought to analyze human pancreatic tissues by scRNA-seq. To allow for contextualization of the cellular patterning and transcriptional activity unique to malignant pancreatic tissues, we included samples of histologically normal pancreas and chronic pancreatitis, in addition to treatment-naïve tumors (untreated PDAC), and tumors from patients who received FOLFIRINOX-based NAT chemoradiotherapy (NAT-PDAC; Supplementary Table S1). Fresh tissue resections were enzymatically dissociated following a protocol that we recently described for tumor stromal cell isolation, and single-cell suspensions were loaded onto a 10x Genomics microfluidics chip for single-cell barcoding and sequenced on the Illumina next-generation sequencing platform (Fig. 1A; ref. 29). In total, 19 individuals (34 total samples) were successfully profiled by scRNA-seq. After filtering out low quality cells (see “Materials and Methods”), 163,275 single-cell transcriptomes were obtained, with roughly equal representation from each sample type (19.6% from normal pancreatic tissue, 26.7% from pancreatitis samples, 26.6% from untreated PDAC, 27.1% from NAT-PDAC specimens). Initial data inspection revealed that samples contained ambient (cell-free) mRNA and therefore each sample was processed with the SoupX algorithm to remove background contamination (Supplementary Fig. S1A–S1D; ref. 31). Transcriptomes were separated into 13 transcriptionally distinct cell clusters (Fig. 1B) and annotated using a large array of widely accepted canonical cell identity genes (Supplementary Fig. S2A). Of note, we did not detect cells with phenotype of tuft cells in our sample preparation.

Differences in the representation of cell clusters were observed according to the sample type (Fig. 1C), which was also mirrored by the overall tissue features depicted by histologic assessment (Fig. 1D). In particular, normal pancreatic tissue was largely comprised of epithelial and endothelial cells (40%), with a limited network of mesenchymal cells (12% fibroblasts and pericytes; Fig. 1E; Supplementary Fig. S2B and S2C). Significant population shifts in tissue composition were observed in PDAC and pancreatitis samples, the most remarkable of which was the dramatic increase in fibroblast representation, highlighting some degree of similarity within the tissue remodeling processes occurring following chronic inflammation and malignant transformation (Fig. 1E; Supplementary Fig. S2B and S2C). Fibroblast cellularity was further exacerbated in patients with PDAC receiving standard-of-care NAT chemoradiotherapy (Fig. 1E; Supplementary Fig. S2B and S2C), likely reflecting a microenvironmental response to therapy. Notably, changes in fibroblast representation across conditions were also mirrored by proportional alterations in gene expression, as shown by the progressive increase in transcriptional variance in fibroblasts from normal to pancreatitis to untreated PDAC, as well as between untreated PDAC and NAT-PDAC (Fig. 1F). Altogether, these data recapitulate the complexity of human pancreas with minimal sample processing and manipulation, supporting the interrogation of the cellular circuitries regulating tissue remodeling in PDAC.

### Ductal cell analysis highlights transcriptional and functional diversity

**Figure 2:**
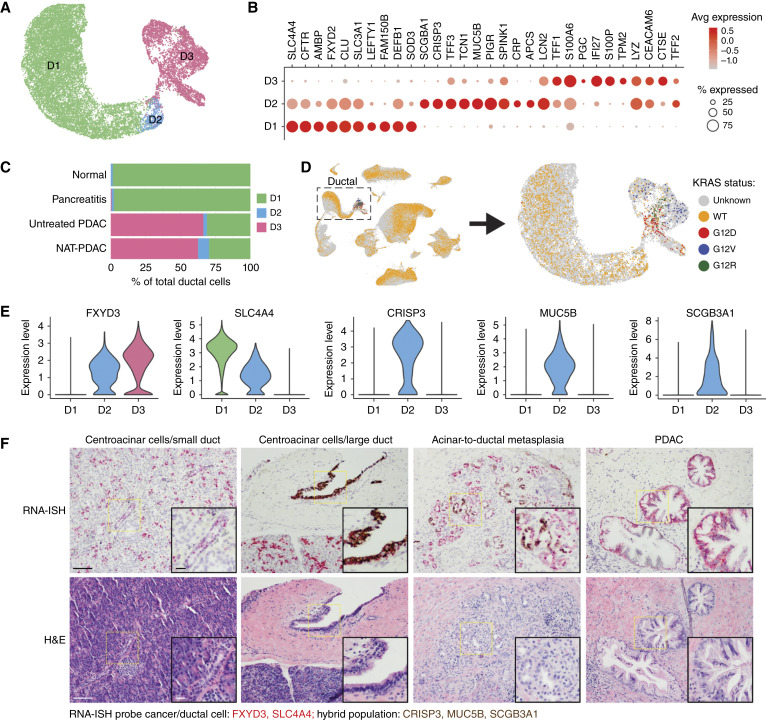
Genetic and transcriptomic features define distinct ductal cell populations. **A,** UMAP projection based on the top five PCs of ductal cell transcriptomes. **B,** Bubble plot demonstrating relative expression of marker genes across clusters. The intensity of the color is proportional to the average expression of the marker within a cluster, and the bubble size is proportional to the number of cells expressing the marker. **C,** Quantification of each cluster within tissue types. **D,** UMAP projections of KRAS mutation status in single cells. Subclustering of the ductal cell population is shown on the right. **E,** Violin plots indicating the expression of marker genes for cancer (*FXYD3*), normal duct (*SLCA4*), and hybrid (*CRISP3*, *MUC5B*, and *SCGB3A1*) cells in the three ductal cell clusters. Expression values in natural-log scale. **F,** Representative images of RNA-ISH (top) and H&E (bottom)-stained section tissue. Composition of RNA-ISH probe cocktails is indicated below. Normal pancreas was used to demonstrate staining of centroacinar cells/small duct (HTB2875) and centroacinar cell/large ducts (HTB2953). PDAC samples were used to demonstrate the staining of acinar-to-ductal metaplasia (HTB2903) and cancer cells (HTB2883). Scale bars, 100 μm for large images and 25 μm for inset images. UMAP, Uniform Manifold Approximation and Projection for Dimension Reduction; H&E, hematoxylin and eosin.

Although ductal cells comprise a relatively small proportion of PDAC (Fig. 1E), they constitute the functional epicenter of the PDAC microenvironment; as such, they bear pivotal information on how tissue imprinting is initiated during neoplastic transformation. For these reasons, the transcriptional programs within the ductal cell compartment across PDAC, pancreatitis, and normal pancreas were further investigated with the aim of identifying distinct functional states associated with tissue repair and malignant transformation. Initial analysis revealed the presence of three major subclusters within ductal cells (18,808 cells analyzed, Fig. 2A). Among these, cluster D1 was found to be enriched for genes indicative of homeostatic functionality of pancreatic ductal cells, including the molecular transporters *SLC4A4*, *CFTR*, *AMBP*, and *FXYD2* (Fig. 2B), and was the predominant subset present in nonmalignant tissues (Fig. 2C). Based on these findings, cluster D1 was determined to account for normal pancreatic ductal epithelial cells. By contrast, cluster D3, which showed an enrichment for markers such as *TFF1* and *S100P* (Fig. 2B), was primarily composed of PDAC samples (Fig. 2C), suggesting that this subset represents cancer cells. Transcriptional profiling of cluster D2 was not sufficient to elucidate the identity of these cells, as they were found to be enriched for genes expressed in both normal ductal epithelial and cancer cells (Fig. 2B). However, these cells were marked by expression of unique genes involved in tissue homeostasis (*MUC5B*), inflammatory (*SCGB3A1*), and cell regeneration programs (*CRISP3*; refs. 46–48).

To further examine the neoplastic nature of ductal cells in each cluster, we developed a companion assay to probe KRAS hot-spot mutations by direct amplification of the regions encompassing amino acids G12 and Q61 from residual cDNA generated as part of the standard 10x Genomics 3′ protocol. By mapping *KRAS* mutation status to single cells, we were able to demonstrate enrichment for *KRAS* mutants in cluster D3 (Fig. 2D), consistent with the malignant phenotype predicted for this subset based on their transcriptional profile. Importantly, mutations in *KRAS* were not found in the other two subsets of ductal cells (clusters D1 and D2; Fig. 2D). These data support the notion that cluster D1 represents normal epithelial cells and suggest that cells in cluster D2 are also not of cancer origin, despite expression of certain cancer gene markers. The relationship of this hybrid D2 cluster to normal and neoplastic ductal cells was further assessed by employing a two-color RNA-ISH assay that allowed for the detection of genes specific to cells in this cluster (*CRISP3*, *MUC5B*, and *SCGB3A1*) as compared with those expressed in PDAC (*FXYD3*) and normal ductal cell (*SLC4A4*; Fig. 2E and F). Using these markers, we were able to probe the spatial localization of cells in cluster D2, revealing their presence in association with large ducts and within a subset of histologically confirmed regions of acinar-to-ductal metaplasia (Fig. 2F). Although a growing body of literature emerges with regard to this novel ductal phenotype, further investigation into its regenerating or tumor-initiating potential is needed to fully define its role in the pancreas microenvironment (46).

To further examine transcriptional differences amongst cancer cells, cluster D3 was subclustered, revealing transcriptional and genomic heterogeneity that suggested the presence of cancer cell subtypes. Limited sample size, however, precludes reliably assessing correlations between the specific *KRAS* variants and cellular phenotypes (Supplementary Fig. S3A–S3D). An apparent enrichment for KRAS-mutant cells carrying the G12D allele was also noted in samples from the treated condition. Unfortunately, there are not enough KRAS-mutant specific samples in this dataset to make correlative statements between KRAS mutants and treatment groups. Remarkably though, cluster heterogeneity within the cancer cell population was less pronounced within the NAT-PDAC sample set (Supplementary Fig. S3E–S3G), suggesting that FOLFIRINOX-based treatment may promote a more transcriptionally homogenous phenotype in cancer cells. Whether this phenomenon stems from universal reprogramming of cancer cells by treatment or from selective survival of those cells with a specific gene signature remains to be determined.

### Inflammatory and tumor-related cues define divergent fibroblast phenotypes

Cellular processes of PDAC cells are sustained by their continuous interaction with the surrounding stroma, and fibroblasts are thought to be the chief organizers of the microenvironmental changes that support tumor progression. However, ambiguity still exists on the specific programs that support fibroblast activation and function. In this context, scRNA-seq can be used to dissect fibroblast phenotypes and states in their natural contexture and discern pathways intrinsically related to tumor progression versus innate programs supporting tissue homeostasis and inflammatory responses.

**Figure 3:**
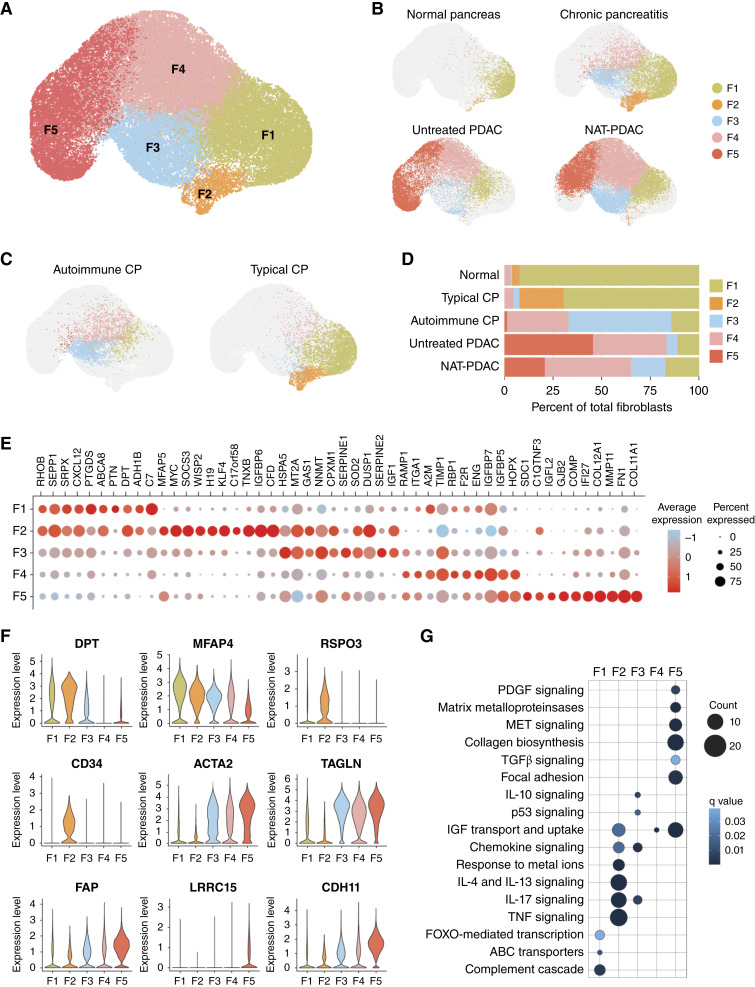
Distinct fibroblast populations are associated with tissue types. **A,** UMAP projection based on the top five PCs of fibroblast single-cell transcriptomes. **B,** UMAP projections of single cells from each tissue type (colored) contributing to the fibroblast UMAP (gray). **C,** UMAP projections of single cells from autoimmune (left) and idiopathic (right) chronic pancreatitis samples (colored) contributing to the fibroblast UMAP (gray). **D,** Quantification of each fibroblast cluster within tissue types. **E,** Bubble plot demonstrating relative expression of marker genes across clusters. The intensity of the color is proportional to the average expression of the marker within a cluster, and the bubble size is proportional to the number of cells expressing the marker. **F,** Violin plots of the expression of genes with defined roles in fibroblast biology. **G,** Visualization of selected KEGG and Reactome pathways significantly enriched in fibroblast clusters. The bubble size is proportional to the number of genes enriched, and the intensity of the color is proportional to significance of the enrichment. Normal fibroblasts (F1); inflammatory fibroblasts (F2); injury-reactive fibroblasts (F3); transitional fibroblasts (F4); CAFs (F5). UMAP, UMAP, Uniform Manifold Approximation and Projection for Dimension Reduction.

To this end, barcoded events annotated as fibroblasts were isolated from all samples and re-clustered, which initially revealed the existence of six fibroblast clusters (Supplementary Fig. S4A). Two of the clusters (clusters 2 and 4; Supplementary Fig. S4A) shared a common tissue origin (PDAC) and showed a striking phenotypic overlap (Supplementary Fig. S4B) and were therefore merged into a single cluster, resulting in five fibroblast clusters, designated as F1–F5 (Fig. 3A). A certain degree of variability in the relative representation of each subset was observed within sample types, suggesting disease-driven functional specialization of mesenchymal cells. For example, although cells within cluster F1 were found to be present in all disease indications, they represented the predominant fibroblast subset in normal pancreas, suggesting that this population largely comprises normal pancreatic fibroblasts (Fig. 3B). A significant shift was observed in PDAC samples including a dramatic expansion of clusters F4 and F5 (Fig. 3B). Relative to untreated PDAC, cluster F3 was notably increased in NAT-PDAC samples, potentially indicating the appearance of a fibroblast subset in response to therapy. Although fibroblasts from pancreatitis samples likewise mapped within cluster F3, these cells were largely contributed by a single patient diagnosed with active autoimmune pancreatitis (Fig. 3C and D), suggesting that cluster F3 represents a more general injury-reactive population induced by chemoradiotherapy or inflammation.

More insights into the functionality of fibroblasts were evinced by analysis of subset-specific phenotypic and functional traits between conditions. For instance, *DPT*, a universal marker of normal fibroblasts, was significantly expressed in cluster F1 (Fig. 3E and F), consistent with the enrichment of this subset in tissues from normal pancreas. *MFAP4*, a gene encoding an extracellular matrix protein of the fibrinogen-related protein superfamily, was also highly expressed in the F1 subset, but downregulated to varying degrees in other clusters (Fig. 3F).

Cluster F2 was characterized by expression of markers implicated with inflammatory processes in fibroblasts, such as *CD34* and *RSPO3* (Fig. 3F), congruent with this cluster predominantly found in patients with chronic pancreatitis (Fig. 3B–D). Pathway enrichment analysis using the genes that distinguished subset F2 further corroborated the influence of inflammatory factors on the state of this fibroblast subpopulation, as demonstrated by enrichment for pathways associated with cytokine and chemokine signaling (Fig. 3G). Notably, this subset also expressed many genes associated with inflammatory CAFs (iCAF; Supplementary Fig. S4C), a population of mesenchymal cells found in neoplastic tissues and predicted to respond to stimuli such as TNFα and IL-1β in the tumor microenvironment (24, 49). Interestingly, cluster F2 was not explicitly associated with malignant tissues but contained fibroblasts derived from both samples of patients with PDAC and patients with pancreatitis (Fig. 3B–D). It is therefore possible that the iCAF cellular program may represent an inflammation-associated functional state arising in injured tissue, irrespective of the presence of malignant cells or cancer signals. Although cellular or molecular drivers of the iCAF signature cannot be definitively ascertained from transcriptomic data alone, the possibility of iCAF-like functional states arising in nonmalignant tissues may warrant future experimental investigation.

Clusters F3, F4, and F5 showed a significant enrichment for genes commonly attributed to myofibroblasts, *ACTA2* and *TAGLN* among others (Fig. 3F). Subset F5 also showed strong expression of well-characterized genes associated with CAFs including *FAP*, *LRRC15*, and *CDH11* (Fig. 3F) as well as canonical functional features of CAFs, such as TGFβ signaling, enhanced extracellular matrix production, and enhanced extracellular matrix remodeling properties (Fig. 3G; refs. 29, 50). These findings are consistent with cluster F5 being found primarily in samples from patients with PDAC and suggest that cluster F5 represents bona fide CAFs. Remarkably, platelet-derived growth factor signaling was also heightened in subset F5 (Fig. 3G), in conjunction with the downregulation of FOXO-mediated transcription, a cellular program that has been linked to suppression of cell proliferation and myofibroblast differentiation (51). As functional inactivation and downregulation of FOXO-dependent pathways through exposure to growth factors such as platelet-derived growth factor have been reported (52), the data support the notion that these inversely regulated pathways may be essential contributing factors governing the proliferative expansion of cells with myofibroblastic features during disease progression. In line with this, genes associated with a specific CAF state known as myCAFs were expressed at high levels in cluster F5 (Supplementary Fig. S4D), indicating that the myCAF phenotype is indeed a predominant cancer-driven state of fibroblast transition in PDAC. The functional identity of cluster F4 was more difficult to ascertain. This subset was largely found in PDAC samples (Fig. 3B–D), expressed high levels of both *ACTA2* and *TAGLN* (Fig. 3F), but only intermediate or very low levels of other CAF markers such as *LRRC15* or *CDH11* (Fig. 3F), raising the possibility that it may represent an intermediate state of fibroblasts transitioning to CAFs.

In light of the features of the herein described subpopulations, the clusters were thereafter referred to as normal fibroblasts (F1), inflammatory fibroblasts (F2), injury-reactive fibroblasts (F3), transitional fibroblasts (F4), and CAFs (F5). Notably, fibroblasts defined here as “CAFs” were not the only fibroblastic cells associated with cancer tissues. However, this naming convention was adapted to capture the conversion of fibroblasts from normal, to an intermediate state, to the mature CAFs that are only found in malignant tissues (whereas clusters F2, F3, and F4 were also found in pancreatitis samples, making these populations not specific to cancer).

### Temporal and spatial differentiation of fibroblasts

**Figure 4:**
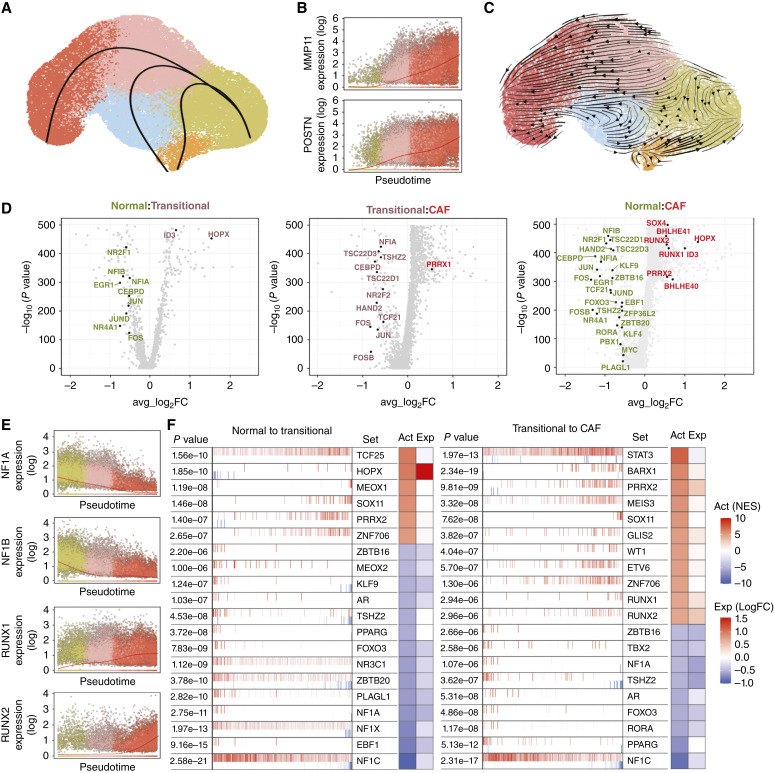
Differentiation of fibroblasts is coupled with a loss of transcriptional regulatory programs. **A,** Trajectory analysis of fibroblast subclusters inferred by Slingshot. **B,** Gene expression of CAF marker genes plotted along the pseudotime trajectory leading to CAFs. Expression values in natural-log scale. **C,** RNA velocity analysis of fibroblast cells. **D,** Volcano plots of DE genes between fibroblast populations. Transcription factors that are DE between populations, with an adjusted *P* value <0.01 and average log_2_FC > 0.5 in absolute value, are indicated. **E,** Gene expression of selected transcription factors plotted along the pseudotime trajectory leading to CAFs. Expression values in natural-log scale. **F,** Top 20 candidate master regulators of transcriptional program altered between fibroblast populations identified by the master regulator analysis algorithm (MARINa). The targets of each transcription factor are shown in vertical bars (repressed genes are in blue, and activated genes in red) and are rank-sorted (*x*-axis) from the one most downregulated to the one most upregulated in the selected conditions: normal fibroblasts compared with transitional fibroblasts and transitional compared with CAFs. The heatmaps on the right side of each panel indicate inferred differential activity (Act) and differential expression (Exp) of the transcription factor. MARINa, MAster Regulator INference algorithm.

To further investigate the relationships amongst fibroblast subsets, we performed pseudotime analysis of transcriptional dynamics to computationally predict likely differentiation trajectories. A finding from this analysis was the existence of divergent trajectories originating from normal fibroblasts (Fig. 4A), with a striking separation between cancer-related and inflammation-driven programs, underscoring the implications for tissue environmental cues in cellular phenotypes and functions. As hypothesized from their phenotype, transitional fibroblasts were positioned at an intermediate point within the CAF trajectory, and their transitional nature along this trajectory was further highlighted by the progressive increase in expression of CAF markers such as *MMP11* and *POSTN* (Fig. 4B). Notably, transitional fibroblasts seemingly fed into the development of injury-reactive fibroblasts as well (Fig. 4B), raising the possibility that a switch may exist at the level of transitional fibroblasts leading to nonoverlapping cell states.

We additionally performed RNA velocity to infer temporal dynamics of each subsets’ transcriptional state (Fig. 4C). As with psuedotime analysis, RNA velocity likewise predicted a directional relationship wherein CAFs arise from transitional fibroblasts. However, the temporal dynamics of injury-reactive fibroblasts predicted by RNA velocity differed from that derived from pseudotime examination, with a predicted transition oriented from this cluster toward either CAFs or normal fibroblasts (Fig. 4C). As injury-reactive fibroblasts are likely associated with chemotherapy-induced injury or inflammation, both of which may be transient states, it is possible that the predicted directionality of RNA velocity is highly influenced by the timing of sample collection relative to treatment and treatment-related tissue effects. As such, interpretation of the temporal or lineage relationship of injury-reactive fibroblasts relative to other subsets by computational means remains challenging and likely requires further experimental studies.

To identify potential regulatory nodes associated with CAF functional modulation, we then compared the expression of transcription factors within fibroblast subsets along the CAF differentiation trajectory (Fig. 4D and E). Strikingly, these analyses revealed a significant downregulation in the expression of many transcription factors associated with fibroblast homeostatic functions in transitional fibroblasts, as compared with normal fibroblasts (Fig. 4D). A similar phenomenon was observed in the transition to CAFs, with most transcription factors expressed in transitional fibroblasts being downregulated in CAFs (Fig. 4D). In line with the pathway enrichment analysis (Fig. 3G), *FOXO3* was among the transcription factors downregulated in CAFs as compared with normal fibroblasts (Fig. 4D), supporting a potential connection between downregulation of FOXO-mediated pathways for the acquisition of the myofibroblastic features that define CAFs. *NR4A1* was also significantly downregulated in the progression from normal fibroblasts to CAFs (Fig. 4D). Notably, NR4A1 has been associated with repression of TGFβ-induced pathways and its expression may reflect the transcriptional programs in normal fibroblasts that counterbalance profibrotic signals to maintain mesenchymal tissue homeostasis (53). Only a few transcription factors were found to be upregulated in CAFs. Among these, *PRRX1* was notable (Fig. 4D), as it has been shown to define a population of mesenchymal cells in wound healing with intrinsic scar-forming potential, raising the possibility that a similar profibrotic program is initiated in PDAC by PRRX1 (54). *RUNX1* and *RUNX2* were also upregulated in CAFs (Fig. 4E). Given their role as key transcriptional drivers of osteoblast differentiation, this finding may support the existence of a common program for collagen-secreting mesenchymal cells that may also be adopted by CAFs in PDAC (55, 56).

Overall, however, the paucity of transcription factors found to correlate with CAFs raises the hypothesis that CAF transition may require transcriptional repression of several key cellular programs, more so than active re-programming. To examine this further, we performed analysis of transcription factor gene regulatory networks using the ARACNe algorithm and then incorporated the MAster Regulator INference algorithm to infer the differential activity of transcription factors between cell populations based on the expression of their associated regulatory gene networks, even when the transcription factor itself is not DE (43, 44). Several transcription factors identified by changes in their expression were also revealed to have reduced (*ZBTB16*, *NF1A*, *TSHZ2*, *FOXO3*, and *RORA*) or increased (*RUNX1*, *RUNX2*, *HOPX*, and *PRRX2*) activity as normal fibroblasts progressed toward CAFs (Fig. 4F). Notably, acquisition of transitional fibroblast features was largely accompanied by inhibition of transcriptional activity for several regulatory pathways, supporting the notion that deviation from normalcy in mesenchymal cells may require repression of basic homeostatic functions (Fig. 4F). As transitional fibroblasts progress to CAFs, additional regulons were found to be downregulated (TBX2 and RORA), together with activation of transcriptional programs such as those regulated by RUNX1 and RUNX2 (Fig. 4F). Whether these phenomena are required and/or sufficient for CAF differentiation remains to be determined; however, these data highlight a very fine balance in the transcriptionally regulated events that altogether allow for CAF differentiation.

**Figure 5:**
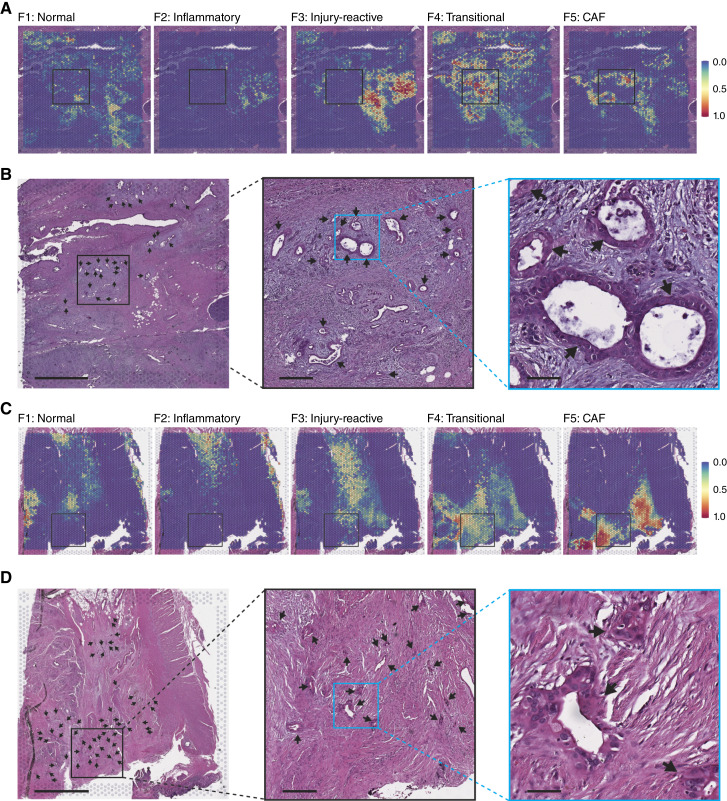
Integration of single-cell and spatial transcriptomics reveals spatially defined fibroblast populations. **A,** Marker gene expression for fibroblast clusters overlayed on untreated PDAC sample HTB2779. Black box corresponds to that in whole mount image in **B**. **B,** Whole mount image and progressively higher magnifications of H&E-stained section of HTB2779. Scale bars, 2 mm (left), 300 μm (middle), and 60 μm (right). The locations of histologically defined cancer glands are indicated by black arrows. **C,** Marker gene expression for fibroblast clusters overlayed on NAT-PDAC sample HTB2903. Black box corresponds to that in whole mount image in **D**. **D,** Whole mount image and progressively higher magnifications of H&E-stained section of HTB2903. Scale bars, 2 mm (left), 300 μm (middle), and 60 μm (right). The locations of histologically defined cancer glands are indicated by black arrows. H&E, hematoxylin and eosin.

Such fine regulation is likely dictated by spatially restricted microenvironmental cues, prompting us to investigate the spatial organization of fibroblast phenotypes. We therefore performed ST analysis from selected PDAC samples to determine whether the fibroblast subclusters identified by single-cell transcriptomics represented spatially identifiable populations. By mapping the transcriptomic signatures identified from the single-cell analyses onto their matched ST data, we identified discrete niches within the tumor microenvironment occupied by fibroblast subsets (Fig. 5A and B showing untreated PDAC sample and Fig. 5C and D showing NAT-PDAC sample). Moreover, although technical constraints of ST limit the extent to which we can define the precise topographical arrangement of subsets within the tumor microenvironment, we nevertheless observed spatial divergence in the progression from normal fibroblasts toward either myofibroblastic or inflammatory phenotypes, mirroring the observations from pseudotime analysis (Fig. 5). Specifically, normal and inflammatory fibroblast subclusters seemingly resided in areas devoid of cancer cells, whereas the myofibroblastic traits of fibroblasts were amplified with increased proximity to cancer cells. These data align with previous findings suggesting that fibroblast subtype polarization may depend on cancer cell–derived cues (57).

### Mapping the pancreas interactome across pathologic states

**Figure 6:**
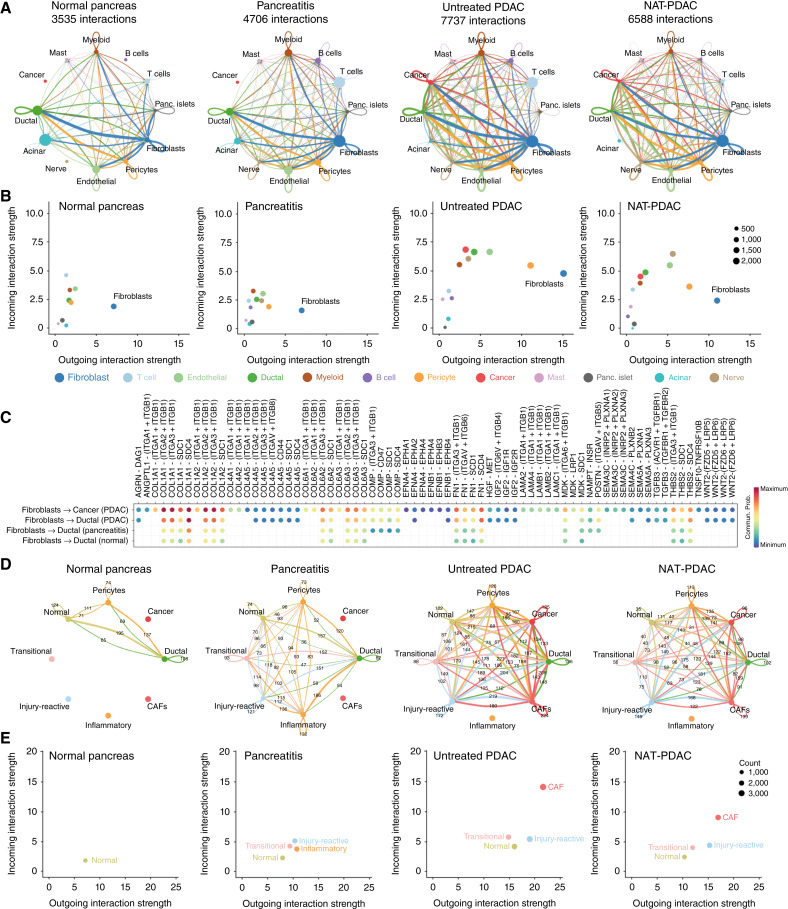
Fibroblasts drive intercellular communication in the tumor microenvironment of PDAC. **A,** Aggregated cell–cell communication network in normal pancreas, pancreatitis, untreated PDAC, and NAT-PDAC samples showing the signaling (edges) sent from each cell population (nodes) inferred using CellChat. The edge weights between any two cell populations are scaled to reflect the total number of inferred interactions. All fibroblast cells are shown as one cell population, to depict the overall interactome driven by the fibroblast compartment as a totality. **B,** Comparison of incoming and outgoing signaling for different cell populations in each tissue type. **C,** Receptor–ligand interaction pairs regulating fibroblasts to epithelial cell communication. **D,** Visualization of inferred cellular communication between mesenchymal and ductal cell populations calculated based on the aggregated cell–cell communication network using CellChat. Each fibroblast subset is separated in this analysis to depict subset-specific interactions. **E,** Comparison of strengths of incoming and outgoing signaling in each fibroblast subset.

The functional heterogeneity of fibroblasts in PDAC, as well as cues involved in temporal/spatial coevolution, reflects the complex network of cellular interactions that support tumor progression. To reconstruct these relationships, we inferred cell–cell communication networks using CellChat, a tool that models the probability of cell–cell communication by integrating gene expression with prior knowledge of the interactions between signaling ligands, receptors, and their cofactors (40). Analyses were visualized using circle plots, to depict the intensity and directionality of interactions. As shown in Fig. 6A and B, prominent signals emanating from fibroblasts (blue) were already notable in normal pancreas samples, underscoring the broad role of mesenchymal cells as obligate structural components of the pancreas. These signals seemed to be directed with higher frequency to normal ductal cells (Fig. 6A), in support of the presence of a mesenchymal–epithelial axis regulating organ homeostasis. Channels of communication between fibroblasts and immune cells were also found (Fig. 6A), in line with the notion that mesenchymal cells possess immunomodulatory potential throughout tissues and conditions (58). A similar scenario dominated by fibroblast-emanated signals was observed in pancreatitis samples, with interesting *de novo* activity toward B cells and nerves (Fig. 6A). The complexity of the interactome was remarkably augmented in PDAC, as displayed by both the number of overall interactions between different cell types, and by their strength (Fig. 6A). Notably, although cancer cells seemed to play a relatively modest role in building these interactions, their activity was found to be primarily directed toward stromal cells, supporting a scenario in which cancer cells may prime the microenvironment by modulating structural elements into reservoirs of cellular signals that sustain cancer progression. Fibroblasts remained the fulcrum of the interactome in PDAC (Fig. 6A and B), showing enhanced communication with partnering cells both through axes already present in benign tissues, as well as through new interaction pathways emerging in the malignant setting (Fig. 6C). The interactive potential of each fibroblast subpopulation was elevated in PDAC, but CAFs dominated these networks of cell–cell communication (Fig. 6D and E). Heightened fibroblast activity seemed to be reinforced by pericytes and endothelial cells as well, suggesting a broad stromal program driving tissue re-programming in PDAC (Fig. 6A and D). Although NAT reduced the predicted communication matrix across all cell types, these effects were most pronounced in the mesenchymal cell compartment (Fig. 6), underlying an important stromal response in PDAC that may have implications for therapeutic responses. The mechanism by which signaling across the post-NAT milieu is blunted remains a critical area for future investigation.

**Figure 7:**
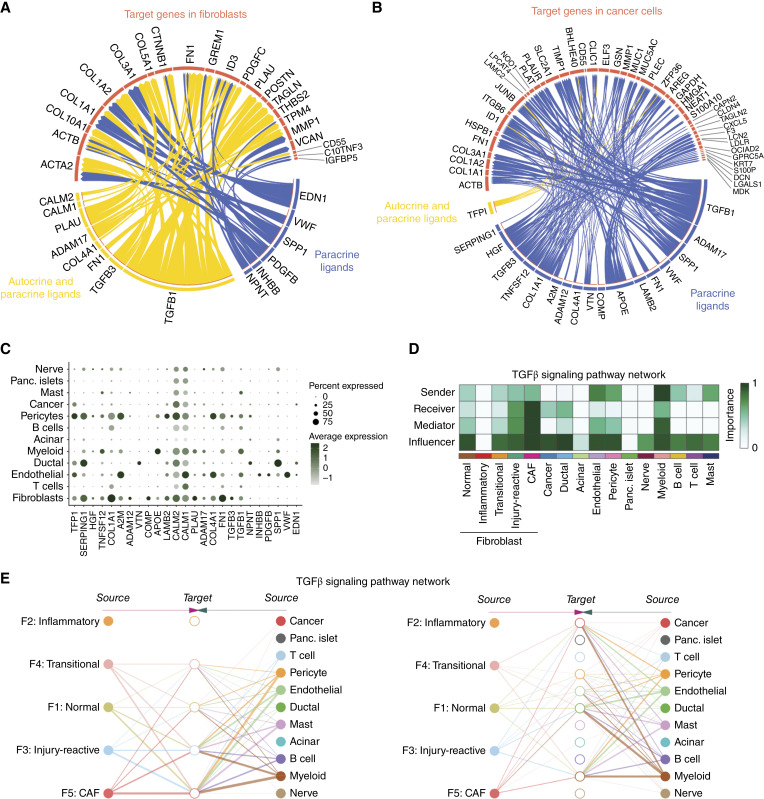
TGFβ is a key mediator of gene expression in both cancer cells and CAFs. **A** and **B,** Circos plots depicting inferred ligand-to-target signaling between target genes (red) in fibroblasts (**A**) and cancer cells (**B**) and their associated paracrine (blue) or autocrine (yellow) ligands identified using NicheNet. Selected target genes represent those enriched in CAFs and transitional fibroblasts from untreated PDAC, relative to normal fibroblasts (**A**) or cancer cells relative to normal ductal cells (**B**). Within each panel, arrow colors indicate the population of cells expressing the ligand and their width indicate the ligand–receptor interaction weights. **C,** Dotplot displaying the expression of ligands identified in **A** and **B** in each cell population. The size of the dot represents the percentage of cells expressing that ligand, and color indicates the average expression level of the ligand across all cells within a cell population. **D,** Heatmap depicting the inferred contribution of cell populations to TGFβ signaling using CellChat. **E,** Hierarchical network diagram visualizing the inferred intercellular communication patterns for TGFβ signaling. Source and target cell populations are represented by solid and open circles, respectively. Line thickness is proportional to the communication probability between cell populations.

Intercellular communication was further interpreted through integration of transcriptomic data with known ligand-to-target signaling paths (41). Using this approach, we mapped unique phenotypic identifiers of CAFs and transitional fibroblasts (relative to normal fibroblasts) to putative corresponding ligands with the goal of highlighting axes of communication contributing to or resulting from fibroblast differentiation (Fig. 7A). Among others, TGFβ proteins stood out as pivotal activators of CAF gene expression. Similar analyses were performed from markers distinguishing cancer cells from normal ductal cells (Fig. 7B). As with CAFs and transitional fibroblasts, TGFβ1 and 3 were likewise highlighted as important paracrine regulators of cancer cells. Notably, cancer cell–specific interactions were largely the result of paracrine signaling, highlighting the critical contribution of TGFβ, together with other factors derived from the tumor microenvironment, in the determination and maintenance of cancer cell programs. Specific cellular sources of associated ligands seemed to vary, although fibroblasts, pericytes, and endothelial cells were notable as abundant producers of the majority of these signals (Fig. 7C). Closer examination of the TGFβ signalosome through CellChatDB highlighted that, despite the presence of a multitude of sources for TGFβ ligands, CAFs and injury-reactive fibroblasts represent prime targets of TGFβ-directed signaling (Fig. 7D and E), consistent with the role of TGFβ in CAF differentiation and permanence (28). Response to TGFβ signaling was notably less prevalent in normal and transitional fibroblast populations (Fig. 7E), suggesting a potential growing reliance on this signaling pathway at later stages of CAF differentiation. Likely, other factors co-act with TGFβ in the modulation of fibroblast phenotypic skew and microenvironment modulation in cancer, as also highlighted by the presence of other CAF-centric networks, including periostin (Supplementary Fig. S5A and S5C) and thombospondin (Supplementary Fig. S5B and S5D).

## Discussion

Genetic instability and cell-intrinsic functional dysregulation are essential features driving cancer initiation and progression; however, the continued growth, survival, and immune evasiveness of tumors are equally reliant on the capacity of cancer cells to co-opt otherwise healthy cellular components of surrounding tissues. Functional insights into the stromal elements of the tumor microenvironment, and the complex web of cellular communication that may underlie their realignment in support of tumor progression, will be essential to the identification of new avenues for therapeutic intervention. Herein, we combined methodologies for the extraction of intact stromal cells from human resections with single cell based and ST analyses to define the microenvironmental dependencies of pancreatic cancer cells, highlighting processes driving differentiation of CAFs and regulatory constituents of the pancreas interactome.

The increased appreciation of how CAFs actively participate in tumor progression and response to therapies has resulted in a spur of explorations aimed at identifying new targets to modulate their functionality. The interrogation of rare stromal cells, however, poses inherent difficulties, stemming from low biomass issues and challenges in their recovery from complex microenvironment with minimal perturbations. Although the implementation of single-cell technologies has provided unprecedented possibilities, sample availability and processing remain problematic. New principles have been derived from analyses of murine models of cancer, highlighting CAF phenotypic and functional heterogeneity (23, 51, 59–62). However, emerging insights from comparative studies suggest that only a handful of CAF transcriptional programs are conserved across humans and mice, supporting the need for broader exploration of these phenomena in human samples (63). In this respect, our study represents a unique and powerful resource to complement recent human cancer datasets, providing access to data from primary pancreatic human samples from a variety of disease states. By making use of state-of-the-art techniques for stromal processing with minimal manipulation, our study furthers our understanding of how mesenchymal cells contribute to the creation of a permissive tumor microenvironment in an unbiased fashion. These technical advantages differentiate our dataset from others generated using pre-enrichment protocols or sorting of rare cells that can otherwise lead to cellular stress and introduction of biases.

A salient point emerging from this study is the appreciation of divergent differentiation trajectories for fibroblasts in association with the organ’s disease state. The striking fate dichotomy between fibroblasts from pancreatitis samples and cancerous tissues in particular highlights how a distinct microenvironmental milieu can differentially imprint on stromal cell functionality in the context of inflammation and cancer. Such programs are seemingly dictated by positional coordinates within tissue anatomical domains, likely reflecting vicinity of stromal cells to cellular sources of specific signaling cues. Conversely, an overall expansion in fibroblasts was observed in both pancreatitis and PDAC, despite the seemingly separate differentiation trajectories, likely reflecting an essential supportive role for these cells in tissue remodeling processes that is independent from the cause of injury. This concept is substantiated by the finding that fibroblasts constitute the fulcrum of the pancreas interactome across different disease states, as demonstrated by their prominent role in establishing interactions with other cellular components of the tissue microenvironment in both normal pancreatitis and PDAC samples. Notably, this fibroblast-centric cellular framework is amplified in the context of PDAC, with significant increases in both the numbers and the magnitude of interactions driven by fibroblast-originated signals. A large proportion of the ongoing signals were found to be emanated by the mature CAFs, consistent with their pivotal role in the tumor microenvironment. However, heightened communication potential was observed in all fibroblast subsets in PDAC, suggesting an overall stimulation of fibroblast activity in the context of cancer and strengthening the rationale for stromal-targeted therapeutics in fibroblast-rich indications such as PDAC.

Remarkably, a significant shift in the phenotype of fibroblasts characterized by simultaneous expression of myofibroblast genes and inflammatory-related features was observed after NAT therapy, supporting that therapeutic intervention, directly or indirectly, may alter the functional attributes of fibroblasts by way of transcriptional reprogramming or subtype plasticity. The extent to which this phenotype may contribute to recalcitrance to therapy in PDAC has yet to be determined. Interestingly, a similar phenotypic profile was abundantly observed in fibroblasts from nonmalignant, autoimmune pancreatitis (but not in other pancreatitis samples), raising the possibility that this cellular subset may result from an acute inflammatory insult. Whether these fibroblasts arise from similar differentiation trajectories remains to be defined.

Another critical aspect underlined by this study is the central role of TGFβ in maintaining the pool of CAFs in the tumor microenvironment, in alignment with previous reports (28, 50, 64). Notably, however, our data suggest minimal dependency for TGFβ signaling in the early phases of fibroblast transition, as highlighted by poor representation of TGFβ-related receptor–ligand interactions depicted in normal and transitional fibroblasts in PDAC. This finding, together with the observation that CAF differentiation is associated with a repression of multiple transcriptional pathways, may indicate that re-programming of fibroblasts in tumors may rely on coordinated activation of desmoplastic responses by TGFβ as well as silencing of tonic signaling controlling homeostatic functions through other yet undefined factors.

It is additionally notable that CAFs, relative to both normal and transitional fibroblasts, seem to be particularly primed for responsiveness to regional signals, as indicated by the enhanced degree of incoming signals inferred by receptor–ligand interaction pairs. Further experimental exploration of such signaling axes may therefore provide valuable insight to microenvironmental features driving CAF function.

The presence of cues from neoplastic cell populations is key to the cell signaling and subtype differences seen between tumor and inflammatory microenvironments. The use of a newly deployed assay for probing KRAS mutation status was instrumental for identifying hybrid ductal epithelial cell, which uniquely shared transcriptional elements with normal ductal and cancer cells. This finding may provide conceptual reasoning for the existence of cellular mechanisms that precede malignant transformation and through which a tumor-supporting microenvironment arises. Although difficult to extrapolate at present, single-cell determination of KRAS variants also offers the opportunity to address essential aspects of PDAC genome instability such as clonality of driver mutations and may provide fundamental insights into the use of mutant-specific inhibitors of KRAS in PDAC. Applying these analytical tools to a larger set of samples may provide important information on the correlation between PDAC cells harboring specific KRAS variants, their transcriptional program, and their microenvironment.

In summary, this study builds upon the accumulating literature surrounding alterations of homeostasis that drive the development of the pancreatic cancer microenvironment. Using single-cell technologies, we describe a new dataset that includes benign and malignant sample types and captures these environmental changes with greater fidelity. Coevolution between cancer cells and stromal elements, namely fibroblasts, emerges as a pivotal axis that distinguishes inflammatory from neoplastic environments and occurs through fibroblast subtype de-differentiation and polarization under cues from neoplastic cell types. Recognizing the interdependent networks between these cellular elements focuses the search on treatments designed to further destabilize tumor circuitry and augment the clinical efficacy of existing regimens. To date, however, relatively few therapeutic efforts aimed at eliminating or restructuring these stromal elements of the TME have gained traction. In this respect, we believe this dataset to be a valuable asset that can be integrated with other available and emerging analyses, providing a rich resource for robust interrogation of stroma-indicative biomarkers and even clinical target identification. Furthermore, although the analyses presented herein focused on fibroblast–cancer cell interactions, this dataset represents an invaluable resource for the assessment of other microenvironmental cellular elements in PDAC, including immune cells, in their natural contexture. Although our methodology for recovering stromal cells from PDAC tissues has allowed analysis of these populations with an unprecedented degree of depth, we must also acknowledge that limited patient numbers may insufficiently represent the totality of patient heterogeneity. Additionally, differences in age and sex distribution across sample type were unavoidable and must likewise be taken into consideration. Nevertheless, our study provides a great resource to the field, building on the emerging bulk of transcriptomic data from PDAC that can be interrogated to inform biomarkers and indicators of stromal fitness underlying pathophysiologic features of the disease.

## Supplementary Material

Supplementary Table 1 Supplementary Table 1

Supplementary Table 2 Supplementary Table 2

Supplementary Figure 1 Supplementary Figure 1

Supplementary Figure 2 Supplementary Figure 2

Supplementary Figure 3 Supplementary Figure 3

Supplementary Figure 4 Supplementary Figure 4

Supplementary Figure 5 Supplementary Figure 5
