# Supplementary material for: Dynamic Evolution of Fibroblasts Revealed by Single-Cell RNA Sequencing of Human Pancreatic Cancer
Source: Cancer Res Commun. 2024 Dec 2;4(12):3049–66. doi: 10.1158/2767-9764.CRC-23-0489 (PMC11609929; doi:10.1158/2767-9764.CRC-23-0489)
Supplement: Supplementary Table 1 [file crc-23-0489_supplementary_table_1_suppst1.pdf]

Supplementary Table 1. Clinicopathologic features of patient cohort

Identifier	*Sample type*	*Age (years)*	*Sex*	*Pathologic stage*[a]	*NAT regimen*	*Pathologic response*	*Disease Etiology*
HTB2779	Untreated PDAC	75	M	T2N1 (IIB)	None	NA[b]	NA
HTB2819	Untreated PDAC	73	M	T2N1 (IIB)	None	NA	NA
HTB2867	Untreated PDAC	68	F	T2N1 (IIB)	None	NA	NA
HTB2883	Untreated PDAC	65	M	T2N0 (IB)	None	NA	NA
HTB2936	Untreated PDAC	88	F	T3N0 (IIA)	None	NA	NA
HTB2840	NAT PDAC	71	F	T2N1 (IIB)	FOLFIRINOX + CRT[c]	Grade 2	NA
HTB2847	NAT PDAC	66	F	T2N0 (IB)	FOLFIRINOX + CRT	Grade 2	NA
HTB2879	NAT PDAC	75	F	T2N0 (IB)	FOLFIRINOX + CRT	Grade 2	NA
HTB2903	NAT PDAC	42	F	T1N1 (IIB)	FOLFIRINOX + SBRT[d]	Grade 2	NA
HTB2905	NAT PDAC	59	F	T2N1 (IIB)	FOLFIRINOX + CRT	Grade 3	NA
HTB3006	NAT PDAC	72	F	T2N0 (IB)	FOLFIRINOX + CRT	Grade 2	NA
HTB2861	Pancreatitis	41	M	NA	None	NA	Alcohol
HTB2862	Pancreatitis	22	F	NA	None	NA	Gallstones
HTB2868	Pancreatitis	58	M	NA	None	NA	Alcohol
HTB2882	Pancreatitis	27	F	NA	None	NA	Iatrogenic
HTB2989	Pancreatitis	61	F	NA	None	NA	Autoimmune
HTB2875	Normal Pancreas[e]	37	F	NA	None	NA	NA
HTB2895	Normal Pancreas	45	F	NA	None	NA	NA
HTB2953	Normal Pancreas	38	F	NA	None	NA	NA

[a]American Joint Committee on Cancer 8th edition; [b]not applicable; [c]chemoradiation therapy; [d]stereotactic-beam radiation therapy; [e]Healthy pancreas collected during resection of MCN
