# Supplementary material for: Dynamic Evolution of Fibroblasts Revealed by Single-Cell RNA Sequencing of Human Pancreatic Cancer
Source: Cancer Res Commun. 2024 Dec 2;4(12):3049–66. doi: 10.1158/2767-9764.CRC-23-0489 (PMC11609929; doi:10.1158/2767-9764.CRC-23-0489)
Supplement: Supplementary Table 2 [file crc-23-0489_supplementary_table_2_suppst2.pdf]

Supplementary Table 2. Experimental conditions for KRAS mutation detection assay.

Primers used for amplification of KRAS-specific sequences

Primer Name	*Sequence (5’->3’)*
Nest 1 & 2 Forward Primer	AATGATACGGCGACCACCGAGATCTACACTCTTTCCCTACACGACGCTCTTCCGATCT
Nest 1 Reverse Primer	GGCCTGCTGAAAATGACTGAATATAAACTTGTGGTAG
Nest 2 Reverse Primer	caagcagaagacggcatacgagatNNNNNNNNgtgactggagttcagacgtgtgctcttccgatctTATAAACTTGTGGTAGTTGG

Breakdown of Nest 2 Reverse Primer

Illumina P7 FC Binding Sequence	*Index*	*Illumina R2 Sequence*	*KRAS Specific Sequence*
caagcagaagacggcatacgagat	NNNNNNNN	gtgactggagttcagacgtgtgctcttccgatct	TATAAACTTGTGGTAGTTGG

PCR cycling conditions for Nest 1

Temperature	*Time*	*Cycles*
95°C	1 min	1
95°C	15 sec	
63°C	15 sec	18
72°C	30 sec	
72°C	3 min	1
4°C	hold	

PCR cycling conditions for Nest 2

Temperature	*Time*	*Cycles*
95°C	1 min	1
95°C	15 sec	18
72°C	1 min	
72°C	3 min	1
4°C	hold	
