# Supplementary material for: Dynamic Evolution of Fibroblasts Revealed by Single-Cell RNA Sequencing of Human Pancreatic Cancer
Source: Cancer Res Commun. 2024 Dec 2;4(12):3049–66. doi: 10.1158/2767-9764.CRC-23-0489 (PMC11609929; doi:10.1158/2767-9764.CRC-23-0489)
Supplement: Supplementary Figure 1 [file crc-23-0489_supplementary_figure_1_suppsf1.pdf]

Supplementary Figure 1

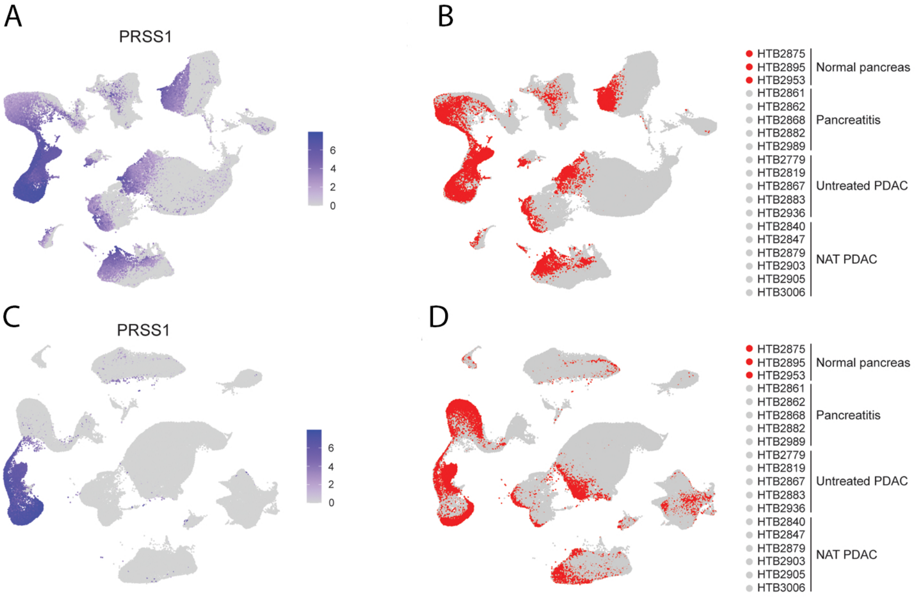

Supp. Fig. 1. Removal of ambient RNA. A. UMAP visualization of single cells from all samples prior to removal of ambient RNA using SoupX. Cells are colored by their level of PRSS1 expression. **B.** UMAP projections of single cells contributing to the global UMAP generated prior to using SoupX. Cells from normal pancreas samples are colored red. **C.** UMAP visualization of single cells from all samples after removal of ambient RNA using SoupX. Cells are colored by their level of PRSS1 expression. **D.** UMAP projections of single cells contributing to the global UMAP after removal of ambient RNA using SoupX. Cells from normal pancreas samples are colored red.
