# Supplementary material for: Dynamic Evolution of Fibroblasts Revealed by Single-Cell RNA Sequencing of Human Pancreatic Cancer
Source: Cancer Res Commun. 2024 Dec 2;4(12):3049–66. doi: 10.1158/2767-9764.CRC-23-0489 (PMC11609929; doi:10.1158/2767-9764.CRC-23-0489)
Supplement: Supplementary Figure 2 [file crc-23-0489_supplementary_figure_2_suppsf2.pdf]

Supplementary Figure 2

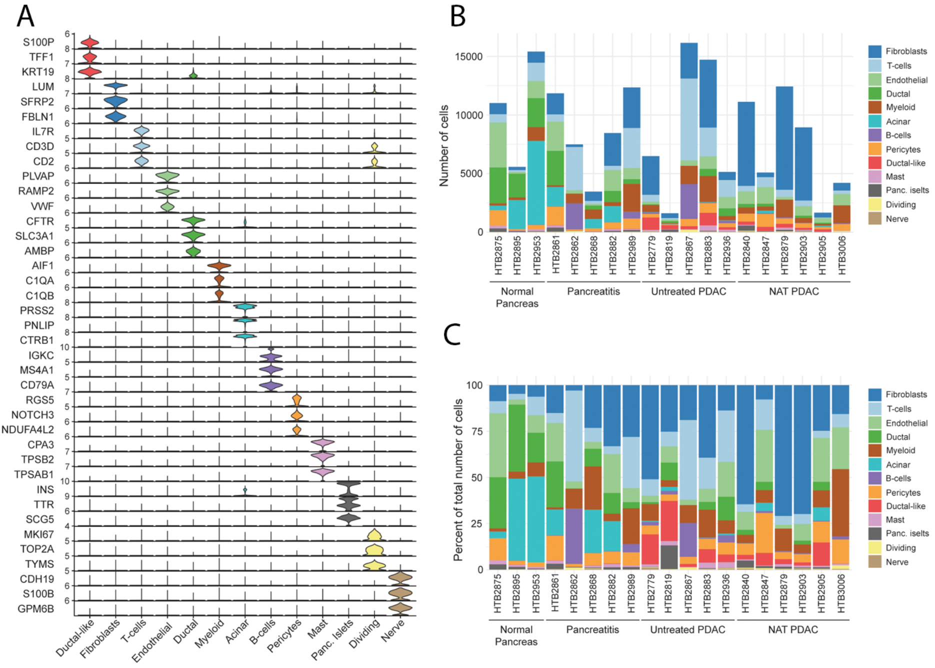

Supp. Fig. 2. Cell lineages in samples analyzed by single cell RNAseq. A. Violin plots of representative genes used to classify transcriptionally defined cell clusters. **B.** Number of cells comprising cell types in each sample. **C.** Relative proportions of cell types in each sample.
