# Supplementary material for: Dynamic Evolution of Fibroblasts Revealed by Single-Cell RNA Sequencing of Human Pancreatic Cancer
Source: Cancer Res Commun. 2024 Dec 2;4(12):3049–66. doi: 10.1158/2767-9764.CRC-23-0489 (PMC11609929; doi:10.1158/2767-9764.CRC-23-0489)
Supplement: Supplementary Figure 3 [file crc-23-0489_supplementary_figure_3_suppsf3.pdf]

Supplementary Figure 3

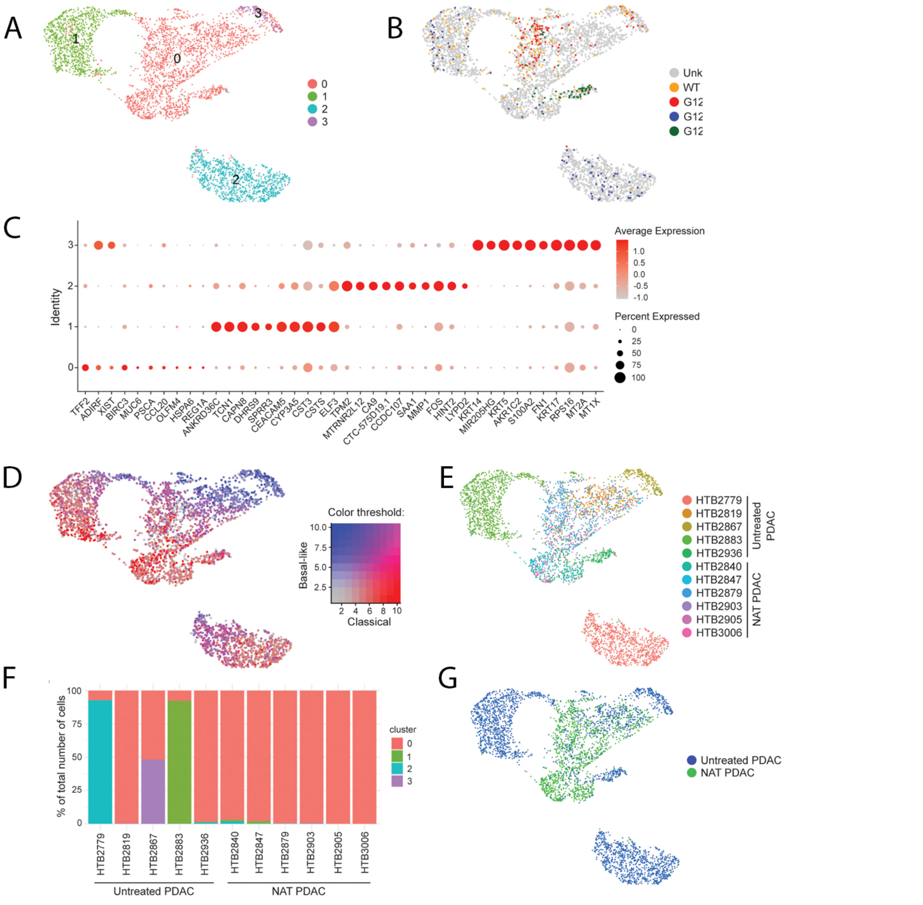

Supp. Fig. 3. Transcriptional and genetic characterization of cancer cells. A. UMAP projection based on the top 5 principal components of cancer cell transcriptomes. **B.** UMAP visualization of cancer cells colored by KRAS mutation status. **C.** Bubble plot demonstrating relative expression of marker genes across cancer cell clusters. The color intensity is proportional to the average expression of the marker within a cluster; the bubble size is proportional to the number of cells xpressing the marker. **D.** UMAP visualization of cancer cells colored by the extent to which individual cells express Classical or Basal-like gene programs. **E.** UMAP projections of cancer cells colored by the contribution of individual samples. **F.** Relative proportions of cancer cell clusters in each sample. **G.** UMAP projections of cancer cells colored by the contribution of untreated and NAT PDAC samples.
