# Supplementary material for: Dynamic Evolution of Fibroblasts Revealed by Single-Cell RNA Sequencing of Human Pancreatic Cancer
Source: Cancer Res Commun. 2024 Dec 2;4(12):3049–66. doi: 10.1158/2767-9764.CRC-23-0489 (PMC11609929; doi:10.1158/2767-9764.CRC-23-0489)
Supplement: Supplementary Figure 4 [file crc-23-0489_supplementary_figure_4_suppsf4.pdf]

Supplementary Figure 4

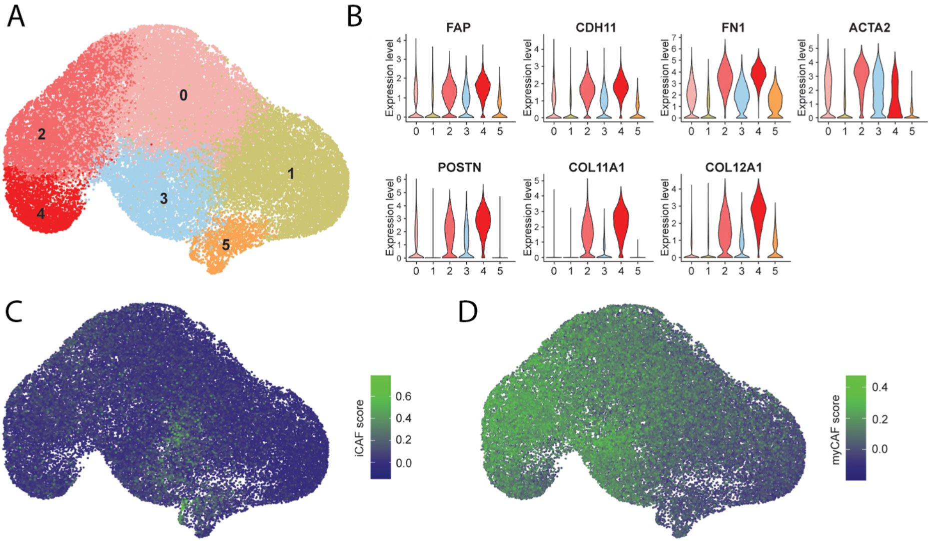

Supp. Fig. 4. Transcriptional profiling of fibroblasts. A. UMAP projection of fibroblasts based on the top five principal components of fibroblast single cell transcriptomes. **B.** Expression of canonical markers of CAFs in each fibroblast cluster. **C.** UMAP visualization of fibroblasts colored by the extent to which individual cells express the iCAF gene program. **D.** UMAP visualization of fibroblasts colored by the extent to which individual cells express the myCAF gene program.
