# Supplementary material for: Dynamic Evolution of Fibroblasts Revealed by Single-Cell RNA Sequencing of Human Pancreatic Cancer
Source: Cancer Res Commun. 2024 Dec 2;4(12):3049–66. doi: 10.1158/2767-9764.CRC-23-0489 (PMC11609929; doi:10.1158/2767-9764.CRC-23-0489)
Supplement: Supplementary Figure 5 [file crc-23-0489_supplementary_figure_5_suppsf5.pdf]

Supplementary Figure 5

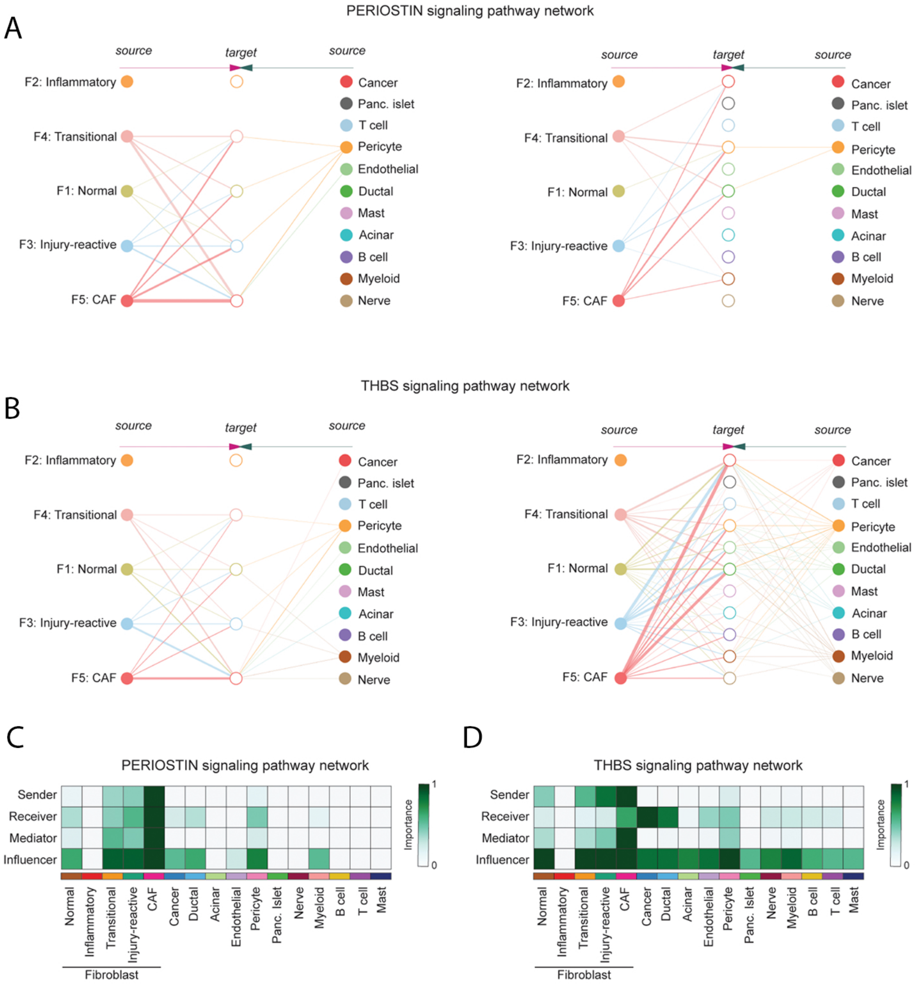

Supp. Fig. 5. Periostin- and Thrombospondin- networks greatly contribute to CAF imprinting in PDAC. A, B. Hierarchical network diagram visualizing the inferred intercellular communication patterns for Periostin (**A**) and Thrombospondin (**B**) signaling. Source and target cell populations are represented by solid and open circles, respectively. Line thickness is proportional to the communication probability between cell populations. **C, D.** Heatmap depicting the inferred contribution of cell populations to Periostin (**C**) and Thrombospondin (**D**) signaling using CellChat.
